# Proteomic Dynamics of Breast Cancer Cell Lines Identifies Potential Therapeutic Protein Targets

**DOI:** 10.1016/j.mcpro.2023.100602

**Published:** 2023-06-19

**Authors:** Rui Sun, Weigang Ge, Yi Zhu, Azin Sayad, Augustin Luna, Mengge Lyu, Shuang Liang, Luis Tobalina, Vinodh N. Rajapakse, Chenhuan Yu, Huanhuan Zhang, Jie Fang, Fang Wu, Hui Xie, Julio Saez-Rodriguez, Huazhong Ying, William C. Reinhold, Chris Sander, Yves Pommier, Benjamin G. Neel, Ruedi Aebersold, Tiannan Guo

**Affiliations:** 1 Westlake Intelligent Biomarker Discovery Lab, Westlake Laboratory of Life Sciences and Biomedicine, Hangzhou, Zhejiang, China; 2 School of Life Sciences, Westlake University, Hangzhou, Zhejiang, China; 3 Key Laboratory of Structural Biology of Zhejiang Province, School of Life Sciences, Westlake University, Hangzhou, Zhejiang, China; 4 Institute of Basic Medical Sciences, Westlake Institute for Advanced Study, Hangzhou, Zhejiang, China; 5 Bioinformatics Department, Westlake Omics (Hangzhou) Biotechnology Co, Ltd, Hangzhou, Zhejiang, China; 6 Institute of Molecular Systems Biology, ETH Zurich, Zurich, Switzerland; 7 Department of Medical Biophysics, University of Toronto, Toronto, Ontario, Canada; 8 Princess Margaret Cancer Centre, University Health Network, Toronto, Ontario, Canada; 9 Laura and Isaac Perlmutter Cancer Center, New York University Langone Medical Center, New York, New York, USA; 10 Department of Biostatistics and Computational Biology, Dana-Farber Cancer Institute, Boston, Massachusetts, USA; 11 Department of Cell Biology, Harvard Medical School, Boston, Massachusetts, USA; 12 Bioinformatics and Data Science, Research and Early Development, Oncology R&D, AstraZeneca, Cambridge, UK; 13 Developmental Therapeutics Branch, Center for Cancer Research, National Cancer Institute, National Institutes of Health, Bethesda, Maryland, USA; 14 Key Laboratory of Experimental Animal and Safety Evaluation, Zhejiang Academy of Medical Sciences, Hangzhou, Zhejiang, China; 15 Faculty of Medicine, Institute for Computational Biomedicine, Heidelberg University Hospital, BioQuant, Heidelberg University, Heidelberg, Baden-Württemberg, Germany; 16 Faculty of Science, University of Zurich, Zurich, Switzerland

**Keywords:** triple-negative breast cancer, data-independent acquisition, proteomics, proteotype, drug perturbation, machine learning, pressure cycling technology, EGFR inhibitor, lapatinib, AKT inhibitor, mTOR inhibitor, everolimus, DUS2, DNMT1, TOP2A

## Abstract

Treatment and relevant targets for breast cancer (BC) remain limited, especially for triple-negative BC (TNBC). We identified 6091 proteins of 76 human BC cell lines using data-independent acquisition (DIA). Integrating our proteomic findings with prior multi-omics datasets, we found that including proteomics data improved drug sensitivity predictions and provided insights into the mechanisms of action. We subsequently profiled the proteomic changes in nine cell lines (five TNBC and four non-TNBC) treated with EGFR/AKT/mTOR inhibitors. In TNBC, metabolism pathways were dysregulated after EGFR/mTOR inhibitor treatment, while RNA modification and cell cycle pathways were affected by AKT inhibitor. This systematic multi-omics and in-depth analysis of the proteome of BC cells can help prioritize potential therapeutic targets and provide insights into adaptive resistance in TNBC.

## Highlights

• Proteomic analysis of 76 breast cancer cell lines and 9 lines with perturbation.
• Machine learning-based multi-omics predicts breast cancer cells’ sensitivity to 90 drugs.
• Longitudinal proteomic profiling provides insights into drug resistance.

## Graphical Abstract

**Figure:**
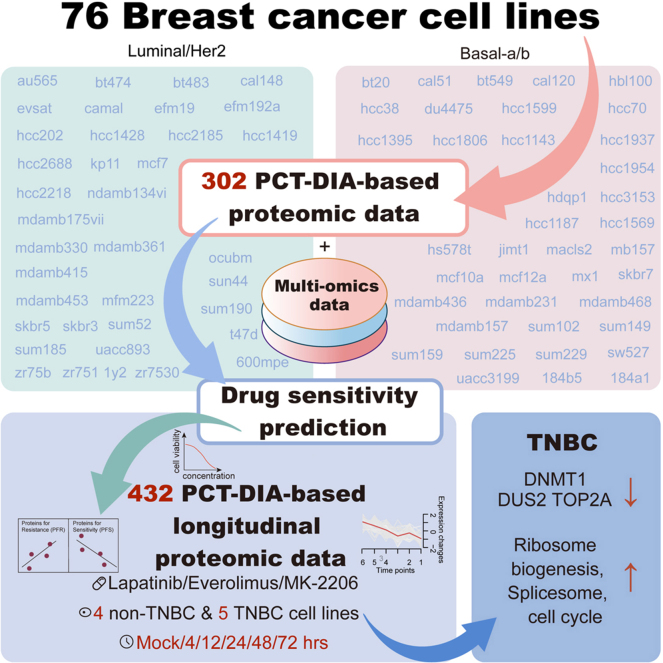

## In Brief

Here we present a deep proteome resource of 76 human breast cancer cell lines. Each cell line included four replicates. Then we built machine learning models using these multi-omics data to predict BC cells’ sensitivity to 90 drugs. We further performed longitudinal proteomic profiling of 9 cell lines perturbed by three drugs. Together, this study presents the so far most comprehensive proteomic investigation of BC cell lines and uncovered novel molecular insights for TNBC cells.

Breast cancer (BC) is the second most common cancer in the world and the most common in women, with a mortality rate ranking fourth among all malignancies (1). In the clinic, BCs are typically classified based on expression of three receptors, namely, estrogen receptor (ER), progesterone receptor (PR), and HER2. Inhibitors targeting ER and HER2 are effective therapeutics in BCs expressing these proteins. However, BCs lacking these receptors are termed “triple-negative” breast cancers (TNBCs). Poly-ADP-ribose polymerase (PARP) inhibitors or immunomodulators, such as antibodies targeting programmed cell death protein 1 (PD-1) and its ligand, are the only targeted therapeutic options for TNBCs but their efficacy has been limited (2). Therefore, most TNBC patients are still treated with conventional chemotherapies, and this subgroup has the worst outcome. Hence, there is an urgent need for developing effective targeted therapies specifically for TNBC patients.

The search for novel drug targets in BC has benefited from multiple well-characterized BC cell lines (3). These cell lines are often divided into four groups based on transcriptomic features: basal A, basal B, luminal, and HER2-positive (4). Luminal lines show high expression of the ER and PR. HER2-positive BC typically features *HER2* (*ERBB2*) gene amplification and high levels of HER2 expression. Basal lines lack ER, PR, and HER2 expression.

Recent genomic profiling studies have revealed potential novel targets for BC (5, 6). However, the pharmacologic accessibility of these targets remains to be established. As proteins are the predominant catalysts of biochemical reactions, they are also the main therapeutic targets (7, 8). Therefore, BC proteomics can complement genomic and transcriptomic studies and provide novel insights into drug response prediction and drug target discovery (8). Cell lines are facile experimental models for drug efficacy tests and simpler model systems than clinical samples or animal models. Indeed, several cancer cell line databases have provided valuable resources for pharmacogenomic studies, such as those from the Cancer Genome Project (CGP) (9), National Cancer Institute (NCI) 60 human tumor cell line anticancer drug screen (NCI-60) (8, 10), the Cancer Cell Line Encyclopedia (CCLE) (11, 12), ProCan-DepMapSanger (13) and the integrated database CellMinerCDB (14). A few studies have analyzed the proteomes of breast cancer cell lines specifically (15, 16, 17, 18). However, these reports utilized small numbers of basal cell lines and focused on unperturbed BC cells or tumors collected at a single time point. By contrast, the response of the BC cell proteome to perturbation by anti-neoplastic drugs has not been investigated, and the mechanisms of acquired drug resistance have not been fully addressed.

Recent advances in data-independent acquisition (DIA)-based quantitative proteomic techniques (19) have enabled high-throughput proteome analysis at considerable depth and a high degree of reproducibility. We further developed a pressure cycling technology (PCT)-assisted semi-automatic tissue lysis and protein digestion technique to minimize technical variation during sample preparation (20).

In this study, we applied our optimized PCT-DIA method to analyze the proteomes of 76 BC cell lines, including 39 TNBC samples. By integrative analysis of our proteomic results with genomic and transcriptomic data, we generated a model to predict the drug response of these BC cells to 90 compounds, including known BC drugs. We then further profiled over 400 BC proteomes from cells perturbed by three drugs that had been prioritized based on our multi-omics modeling of drug response. This unique proteomic dataset allowed us to identify proteins whose expression patterns change in response to these drugs, as well as potential biomarkers for drug resistance in TNBC.

## Experimental Procedures

### Experimental Design and Statistical Rationale

The first part of this study is the generation of the BR76 proteomic dataset where we collected 302 proteomic raw files for 76 BC cell lines. Each cell line was analyzed with three biological replicates and the third biological replcates was further subjected to one technical replicate. This BR76 proteomic dataset was then combined with the published genomic and transcriptomic datasets (5) to predict drug response using elastic net analysis. The other dataset we generated in this study is the BRP dataset, which comprises a perturbation proteomic dataset of nine BC cell lines treated with three drugs (EGFR/Akt/mTOR inhibitors) at different time points. This dynamic proteomic dataset, containing 432 proteomic raw files, captures the proteomic changes over time (0, 24, 48, and 72 h) following drug treatment in nine BC cell lines. We investigated the drug response for each cell line presented as IC50, the dynamic change of protein expression in response to treatment (multiple time points), respectively, using Pearson correlation and Mfuzz analysis. Statistical tests included Benjamini-Hochberg (B-H) adjusted two-tailed unpaired Student’s *t* test or one-way ANOVA for differentially expressed analysis and Pearson correlation test for correlation analysis, which are described in figure legends and methods where relevant.

### PCT-DIA Analysis of the Cell Lines

PCT-DIA analysis was performed as previously described (21). Fresh cell lysates were processed according to a previously published protocol (20). All samples were spiked with iRT peptides (Biognosys) (22). A total of 1.5 μg of cleaned peptides was injected and separated by Eksigent Nano LC 415 (with a 1–10 μL/min flow module to switch the LC from nano-flow to micro-flow. Buffer A: 2% ACN, 0.1% formic acid (FA), buffer B: 98% ACN, 0.1% FA, 5% to 32% linear gradient over 60 min with a flow rate of 5 μL/min) combined with SWATH-MS on a TripleTOF 5600+. Eksigent analytical column (0.3 × 150 mm C18 ChromXP 3 μm) and trap column (0.3 × 10 mm, C18 ChromXP 5 μm) were used for chromatographic separation as previously described (23). The ion accumulation time for the MS1 and MS2 acquisition was set to 150 ms (400–1200 *m/z*) and 30 ms for each window, respectively. The DIA window schemes were optimized to 66 variable windows. The instrument was operated in a high-sensitivity mode.

### DIA Data Analysis

The DIA MS raw data were processed using the DIA-NN (1.7.15) against an established spectral library that contains 194,899 proteotypic peptide precursors and 10,323 proteotypic SwissProt proteins (PRIDE project PXD009597, released on October 21, 2019) (8). DIA raw data files were firstly converted in profile mode to mzML using msConvert, then the mzML files were analyzed using DIA-NN, as described previously (24). Detailed parameters are set as follows. The protease was trypsin/P and the maximum missed cleavage was 2. Modifications included N-term Met excision, Cys carbamidomethylation, and Met oxidation. The peptide length range was set from 7 to 30 amino acids. The m/z range for precursor ions and fragment ions was set from 400 to 2000, 100 to 2000 respectively. The FDR cutoffs of precursor ions, peptides, and proteins were all set at 1%. We used the single-pass mode of the neural network class.

In total, 8952 proteins were identified with a 54.6% missing rate of the whole proteome. We then removed two outlier samples with extremely low protein identification numbers as revealed by PCA plots. After further removing the proteins with over 90% missing rate, 6091 proteins remained, with a 34.7% missing rate of the whole data set. In the meantime, the batch effect across all samples and the reproducibility of the measurement were evaluated by PCA plots and the coefficient of variation of all biological and technical replicates (supplemental Fig. S1).

### Elastic Net Analysis

We used a flexible network algorithm to develop a multivariate linear model to predict the effect of each compound in 76 BC cell lines using genomics, transcriptomics, and proteomics datasets, as previously described (8).

The “glmnet” R package was utilized for elastic network analysis, incorporating nested cross-validation. Our analysis included the following aspects: point mutations (123 mutations), RNAseq (29,140 mRNAs), proteomic data sets (6091 proteins), and RPPA (218 analytes), which were used as input. The “impute.knn” function in the Bioconductor imputes the missing values.

The detailed processes were outlined below. First, an “internal” cross-validation was performed. In this step, a set of multi-omic data from 59 cell lines was employed to determine the optimal hyperparameters (α and λ) for the elastic network. The elastic network parameters α and λ were selected by minimizing cross-validation errors within the “internal” pipeline (average of ten replicates of 10-fold cross-validation). The selected α and λ parameters were then applied to the run of the 200 elastic network algorithms, with each time using a random subset of data obtained from 90% of the available cell lines. Then, the obtained 200 coefficient vectors were averaged, and the predicted values were sorted according to the magnitude of the average coefficient weight. To select predictors suitable for application to new data of finite size, standard linear regression and 10-fold cross-validation are used to evaluate the set of pre-k-ary predictors (by averaging coefficient weights). The cross-validation error within one standard deviation of the minimum cross-validation error, k, was set to a minimum. As part of the “external” pipeline, leave-one-way cross-validation is applied throughout the process to obtain a reliable estimate of the performance of the new data. Specifically, the elastic network model obtained using the remaining data (and the above steps) predicted the drug response of each cell line. Finally, the predicted response value was correlated with the actual response value, and for any drug/data combination, the prediction accuracy was evaluated using the Pearson correlation between the observed and the predicted drug responses (supplemental Table S2).

### Cell Culture

BT549, Hs578T, ZR75, and MDA-MB-231 cell lines were cultured in DMEM/F-12 medium (Biological Industries); MDA-MB-468 was maintained in L15 medium (Biological Industries); T47D was adapted in DMEM medium (Biological Industries); MCF7, MX-1, and SK-BR-3 were kept in 1640 medium (Biological Industries). These media were all supplemented with 10% fetal bovine serum (Biological Industries) and penicillin-streptomycin (HyClone) and incubated at 37 °C with 5% CO_2_.

### Cell Proliferation Assay

Half-maximal inhibitory concentration (IC_50_) was determined using the MTT cell proliferation assay following the manufacturer’s recommendations (25). Briefly, 5000 cells were plated in each well of a 96-well plate with 100 μL medium. After 24 h, the drug gradient concentration was replaced, including lapatinib (Targetmol, 231277-92-2), AKT1-2 inhibitor MK-2206 (Targetmol, 1032350-13-2), and everolimus (Targetmol, 159351-69-6). The cells were then incubated for 72 h. Untreated cells were used as negative controls. The wells without cells were used as blank controls. Next, 20 μL of the 5 mg/mL MTT reagent (Sigma) was added to each well, and the plates were incubated for an additional 4 h at 37 °C. The remaining MTT solution was removed, and 100 μL DMSO (Biofroxx) was added to dissolve the purple formazan and lyse the cell to release the mitochondrial residues of formazan. Absorbance measurements were performed at 570 nm, and the IC_50_ values (*i.e.*, the concentrations that inhibit cell proliferation by 50%) were obtained using Multiscan Spectrum (BioTek).

### Drug Treatment

Cells were treated to different drugs, at their respective IC_50_ concentrations for 4, 12, 24, 48, and 72 h. Following drug exposure, cellswere then washed three times with PBS and collected by centribugating at 2000 rpm for 5 min. After discarding the supernatant, the dry pellets were preserved at −80 °C.

### Opposite Dynamic Proteins Selection

The proteins were clustered in TNBC and non-TNBC cell lines using Mfuzz (26). We only focused on proteins that: (i) consistently up- or down-regulated over time (B-H adjusted ANOVA *p-value* < 0.05) with every drug and (ii) showed a fold-change greater than two between the last and first time points. We then searched for pathways including these proteins using KEGG pathways in STRING database. Prioritization was given to pathways that were consistently dysregulated in at least three out of five TNBC cell lines and at least two out of four non-TNBC cell lines.

## Results and Discussion

### Proteotyping of 76 BC cell Lines

**Fig. 1:**
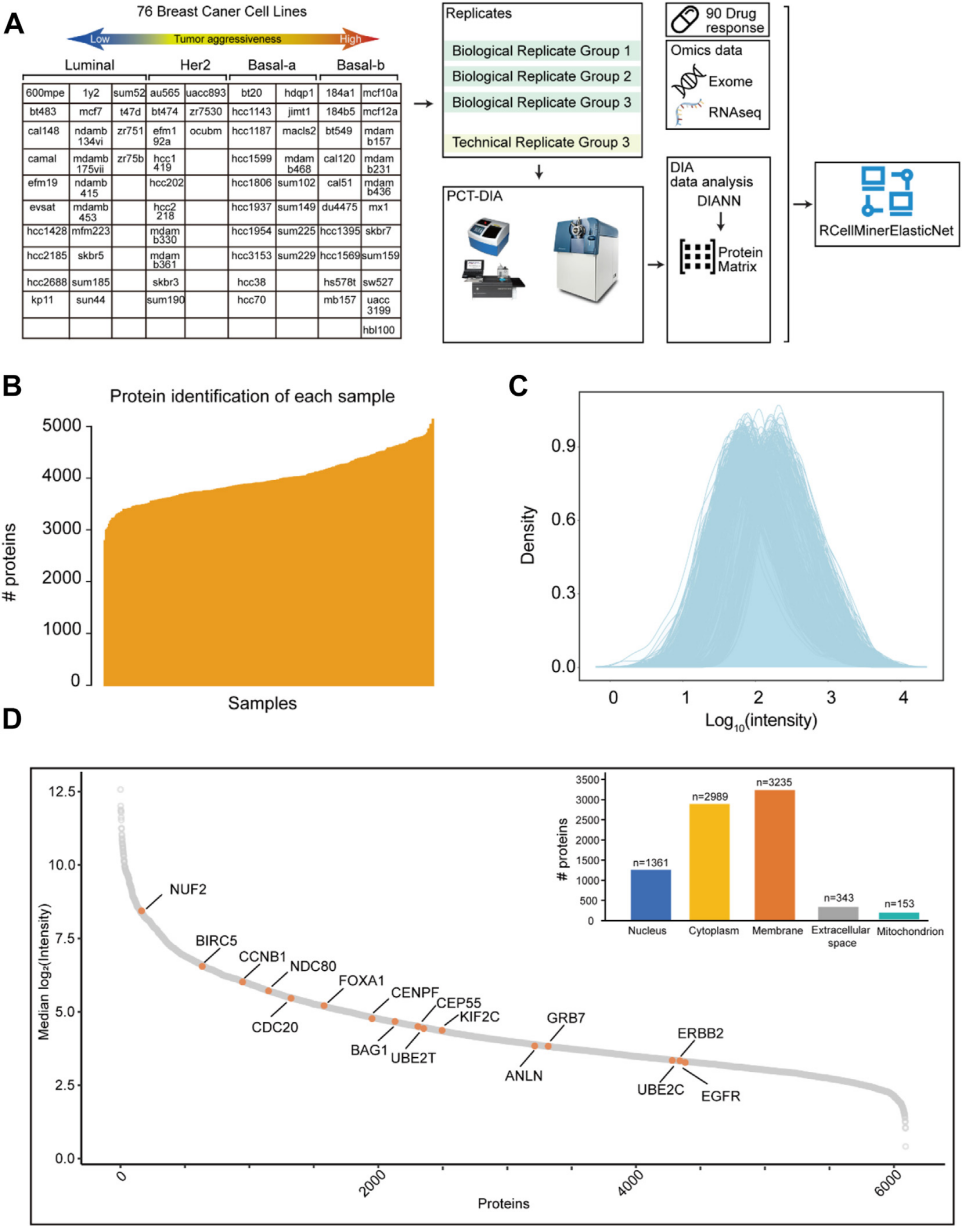
**Proteomics analysis of 76 breast cancer cell lines.***A*, workflow for generating the proteomics dataset of 76 breast cancer cell lines. The cell lines are classified into four subtypes according to the Neve classification (4): basal-like A (basal-a), basal-like B (basal-b), luminal, and HER2-enriched (Her2). Four replicates of each cell line were analyzed using PCT-DIA. The proteomics data were integrated with exome and RNAseq data to predict the drug response of these cell lines to 90 drugs using rcellminerElasticNet. *B*, number of identified proteins in each sample. *C*, density plot of the quantified protein intensities. *D*, ranking of identified proteins according the median of log2-scaled protein intensity. *Orange*-labeled proteins were overlapped with PAM50.

We collected the 76 BC cell lines (5) characterized in our previous BC functional genomics study and classified them into four subtypes, namely luminal, HER2+, basal A, and basal B as in the previous study (4, 5) (Fig. 1*A*). This panel in our study shares 29 lines with the CCLE proteomics study (31 BC cell lines) (11), 44 lines with the ProCan-DepMapSanger resource (50 BC cells) (13). Only two lines from the CCLE study are missing in our panel: CAL851 and HCC1500. Furthermore, 45 of 51 cell lines from the Genomics of Drug Sensitivity in Cancer collection (27) are included in our data set. The proteomes of our 76 BC cell lines were acquired by optimized PCT-DIA technology (20, 28). While other large-scale quantitative proteomic studies analyzed limited replicates (15, 16, 17, 18), our study benefits from the high-throughput nature of the PCT-DIA technique. This permits the analysis of a larger number of samples and yields high-quality data. For each cell line, we analyzed four samples, including three biological replicates and a technical replicate of the third biological replicate. A total of 302 DIA maps (supplemental Table S1*A*) were obtained after quality filtering. Using stringent quantitative criteria as described in the Experimental Procedures section, we consistently identified 90,762 proteotypic peptides from 6091 SwissProt proteins (supplemental Table S1*B*) across all samples with a 34.7% missing rate of 6091 proteins quantified in all samples. For each sample, we quantified an average count of around 4000 proteins (Fig. 1*B*), and the log10 transformed peptide intensities showed a normal distribution (Fig. 1*C*).

**Fig. 2:**
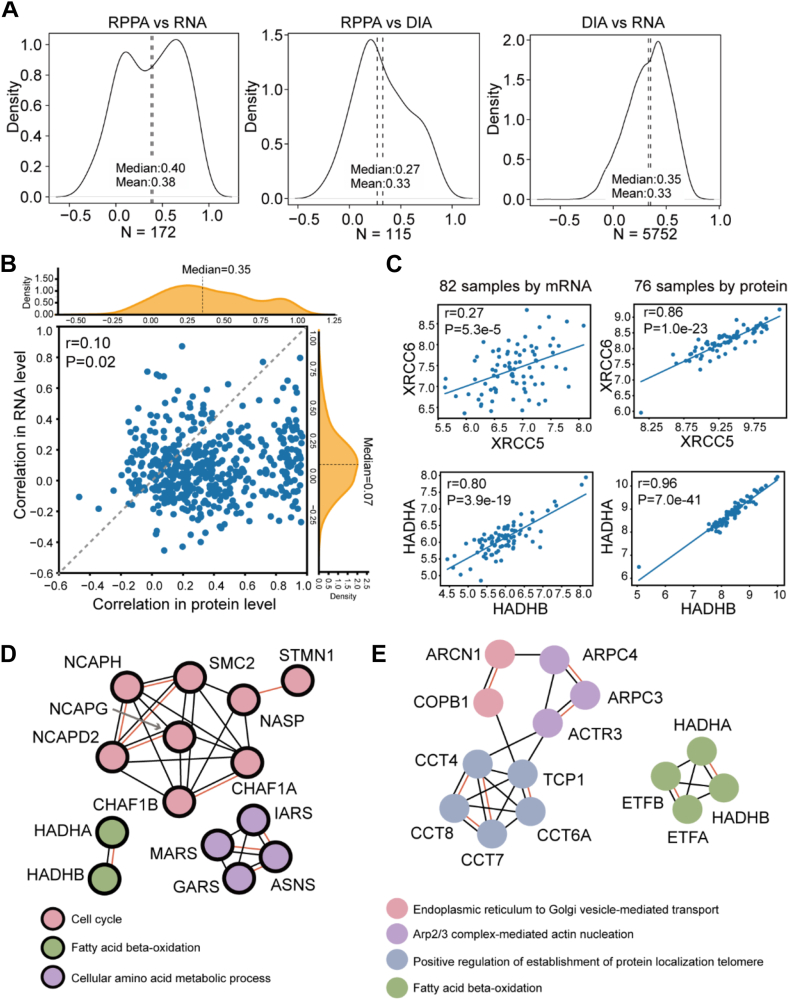
**Multi-omics data comparison.***A*, density plots of the Pearson correlation coefficients resulting from the pair-wise comparisons between the DIA, the reverse phase protein arrays (RPPA), and the transcriptomics datasets. *B*, Pearson correlation of protein complexes between the transcript level and protein level. The statistical significance of the difference between the transcript and protein levels was evaluated by a two-tailed unpaired Student’s *t* test. *C*, expression of the protein complexes XRCC6-XRCC5 and HADHA-HADHB at both the protein and transcript levels. *D* and *E*, network analyses of the ten protein complexes most highly represented at the transcript (*D*) and the protein (*E*) levels using STRING.

Replicate proteomic analyses enabled rigorous assessment of quantitative accuracy and batch effects. Samples from Groups 1 to 3 were biological replicates, while samples from the fourth group served as technical replicates of Group 3, but were processed with a different mass spectrometer. The data quality evaluation of these samples based on the replicates is shown in supplemental Fig. S1. The results indicated that all the replicates had a similar distribution of peptide length with over 95% of the peptides consisting of 7 to 22 amino acid length coefficient of variation (CV) < 10%, supplemental Fig. S1*A*). All replicates consistently yielded comparable protein and peptide identifications. The numbers of peptides identified per protein were highly consistent across the replicates with a CV <10% (supplemental Fig. S1*B*). Regarding protein quantification, the correlation among technical replicates (median *r* = 0.93) was slightly higher than that among biological replicates (median *r* = 0.91), and much higher than that among non-replicate samples (*r* = 0.74). The median CVs across different abundance level were all lower than 0.2 (supplemental Fig. S1*C*). Next, we subjected these proteins for the downstream analysis. The protein intensity distribution identified from these DIA files was highly consistent (supplemental Fig. S1*D*). Principal component analysis (PCA) revealed a mild batch effect in signal intensities in group 4 only, probably because these samples were analyzed by a different mass spectrometer (supplemental Fig. S1*E*). PCA plots of the cell line proteomic data exhibited minimal batch effect after batch correction using “limma R” package (supplemental Fig. S1*F*). Within the dataset, we identified 16 proteins overlapping with the PAM50 gene panel, which is a well-known breast cancer classifier (Fig. 1*D*). We then computed the average protein intensities of the four groups for subsequent analyses. Comparing the gene-wise correlation of RNA-seq (supplemental Table S1*D*) (5), reverse phase protein arrays (RPPA) (supplemental Table S1*C*) (5), and our DIA datasets, we found a slightly low correlation between proteomic and transcriptomic data (Fig. 2*A*), which agrees with previous observations (29). The mRNA data and RPPA data are from a previous publication and not directly derived from the same cell pellets used for our proteomics analysis. The correlation between the protein and mRNA expression is slightly lower than that reported from multi-omics data sets (30, 31, 32) which reports a Pearson correlation of 0.4∼0.5 in tumor tissues. This is probably due to the genetic variability of cell lines propagated across different labs and subcultures (33). Should the same cell pellets be used for both transcriptomics and proteomics analysis, the correlation might be higher. We also examined multiple genes of relevance to BC progression, such as *ERBB2, YWHAE, YWHAB, YBX1, VDAC1,* and *STMN1*. They showed diverse patterns of correlation among the three data sets (supplemental Fig. S2).

### Detection of Protein Complexes in BCs

The investigation of protein complexes could provide new insights into the future development of targeted therapies for BC (34). Using a reference of the complex list containing 622 putative human soluble protein complexes list (35), we matched 487 known complexes containing any two subunits in a complex following the methodology as described previously (8). Consistent with an earlier report (36), the Pearson correlation between proteins within a complex pair (median = 0.35) was significantly higher than that between their cognate transcripts (median = 0.07), as indicated by the more remarkable shift to the right shown in Figure 2*B*. Then we analyzed the top ten protein complex pairs with the highest correlation at both protein and transcription levels. They exhibited a higher correlation at the protein level than that at the transcription level (supplemental Fig. S3). The top ten protein complex pairs at the protein level involved in the cell cycle, fatty acid beta-oxidation, and cellular amino acid metabolic processes (Fig. 2*D*), while that at the transcript level were associated with the processes such as endoplasmic reticulum to Golgi vesicle-mediated transport, actin nucleation, protein localization and fatty acid beta-oxidation (Fig. 2*E*). Notably, fatty acid beta-oxidation was the common pathway detected at both protein and transcription levels. Two exemplar protein complexes (Fig. 2*C*) include the HADHA/B which are potential prognostic biomarkers for BC (37), and the XRCC6-XRCC5 complex involved in DNA damage repair (38). Similar differences in the correlation of the complex component abundances at protein and transcription levels were also apparent in colon cancer (39). Therefore, phenotype-related functions may be better reflected at the protein level.

### Identification and Validation of 38 Proteins Differentially Expressed in Basal Cell Lines

**Fig. 3:**
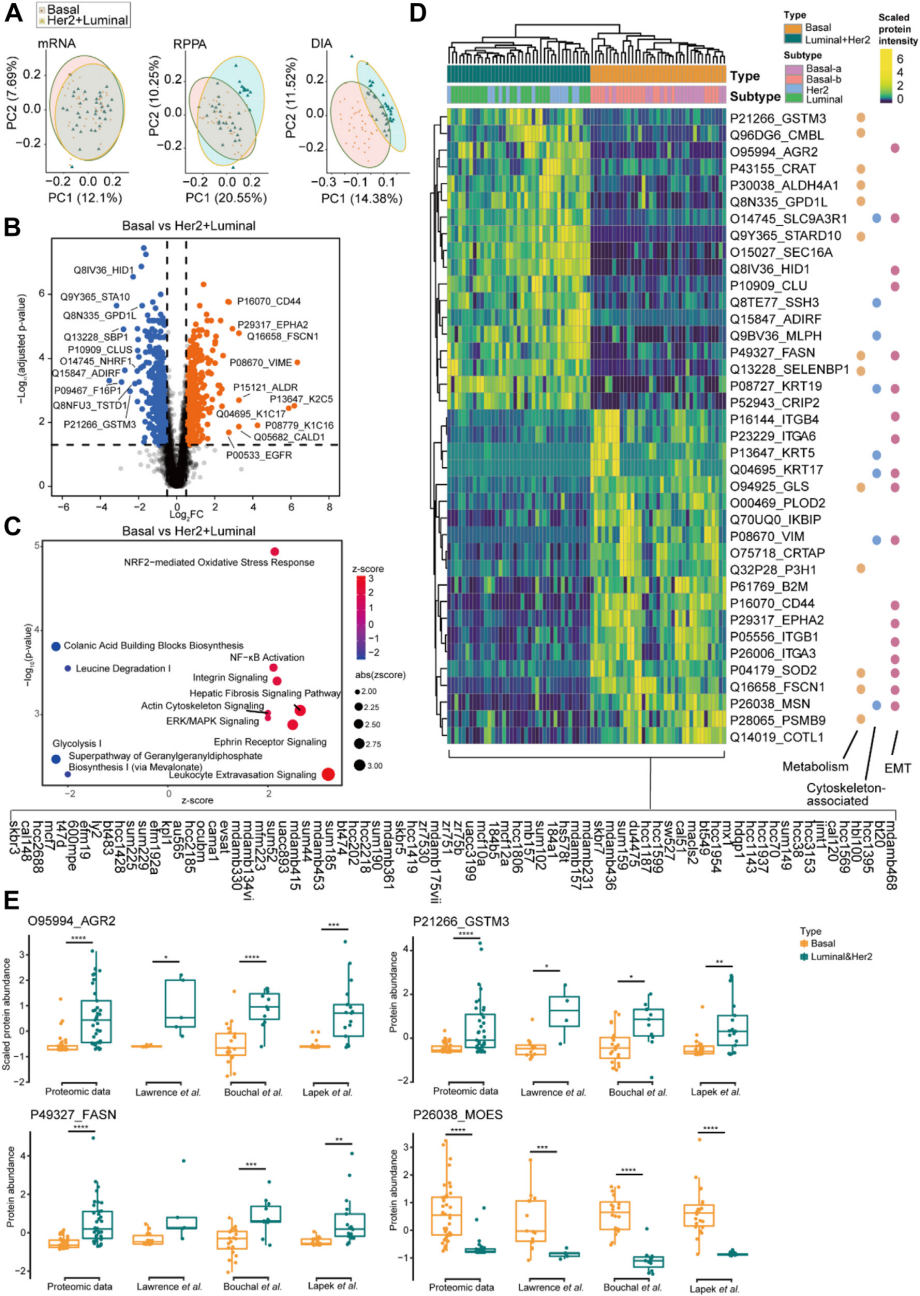
**Differentially expressed analysis between TNBC and non-TNBC cell lines.***A*, PCA plots of the transcriptomics, RPPA, and DIA datasets of TNBC and non-TNBC samples using all identified genes/proteins. *B*, volcano plot of the differentially expressed proteins (DEPs) between TNBC and non-TNBC cell lines (B-H adjusted *p-value* < 0.05, fold change > 1.5 or < 0.67). *C*, bubble plot showing the differentially activated pathways as analyzed by IPA. *D*, heatmap showing the hierarchical clustering analysis of 38 selected protein expressions across the 76 cell lines. *E*, protein expression of AGR2, GSTM3, FASN, and MOES as measured by our proteomics analysis, as well as by Lawrence *et al.* (50), Bouchal *et al.* (17), and Lapek *et al.* (15).

In analyzing molecular patterns unique to TNBC cells, we found that the DIA data outperformed RNA-seq and RPPA datasets in distinguishing TNBC samples from the 76 cell line samples based on 29,140 transcripts, 218 proteins identified by RPPA and 6091 proteins identified by DIA, respectively (Fig. 3*A*). Of note, this analysis was based on all molecules measured in each dataset. Although the discriminative power of the transcriptome is not optimal because of the inclusion of the entire transcriptome containing 29,140 transcripts, the selection of differentially expressed transcripts (DETs, n = 6572) could well resolve the two groups (supplemental Fig. S4*A*). We identified 771 differentially expressed proteins (DEP)s in TNBC *versus* non-TNBC cell lines (Fig. 3*B*). Those DEPs were significantly enriched in 12 differentially activated pathways using Ingenuity Pathway Analysis (IPA) (Fig. 3*C*), including extracellular matrix, metabolism, and immunity network relationship. We also compared with the proteomic data of BC patients (40), identifying 96 overlapping proteins distinguish patients with TNBC from those with non-TNBC patients (supplemental Fig. S4, *B* and *C*). The data indicated that proteome data obtained from cell lines are also potentially informative to the clinic.

We next selected the 38 most significant DEPs to clearly distinguish TNBC cell lines (Fig. 3*D*). These 38 DEPs participated in multiple biological processes (Fig. 3*D*) that are known to be involved in BC progression (41, 42). In particular, 47.4% of the 38 DEPs are markers of the epithelial–mesenchymal transition (43), indicating that they might contribute to BC. Among these proteins, several have been reported to be associated with BC prognosis, such as estrogen-regulated anterior gradient 2 (AGR2) (44), fatty acid synthase (FASN) (45), glutathione S-transferases mu3 (GSTM3) (46), and clusterin (CLU) (47). These proteins were downregulated in the basal groups (Fig. 3*E*). Additionally, FASN (45), ITGB4 (48), and ITGA6 (49) have been identified as therapeutic targets in BC. We also confirmed the applicability of the 38 proteins for BC stratification in three independent breast cancer proteomic datasets (15, 17, 50) (supplemental Fig. S4, *D*–F).

### Proteotyping Improves Drug Response Prediction in Multi-Omics-Based Modeling

Next, we asked whether proteomic data could improve the multi-omics prediction of drug response in TNBC and identify potential biomarkers of drug sensitivity.

**Fig. 4:**
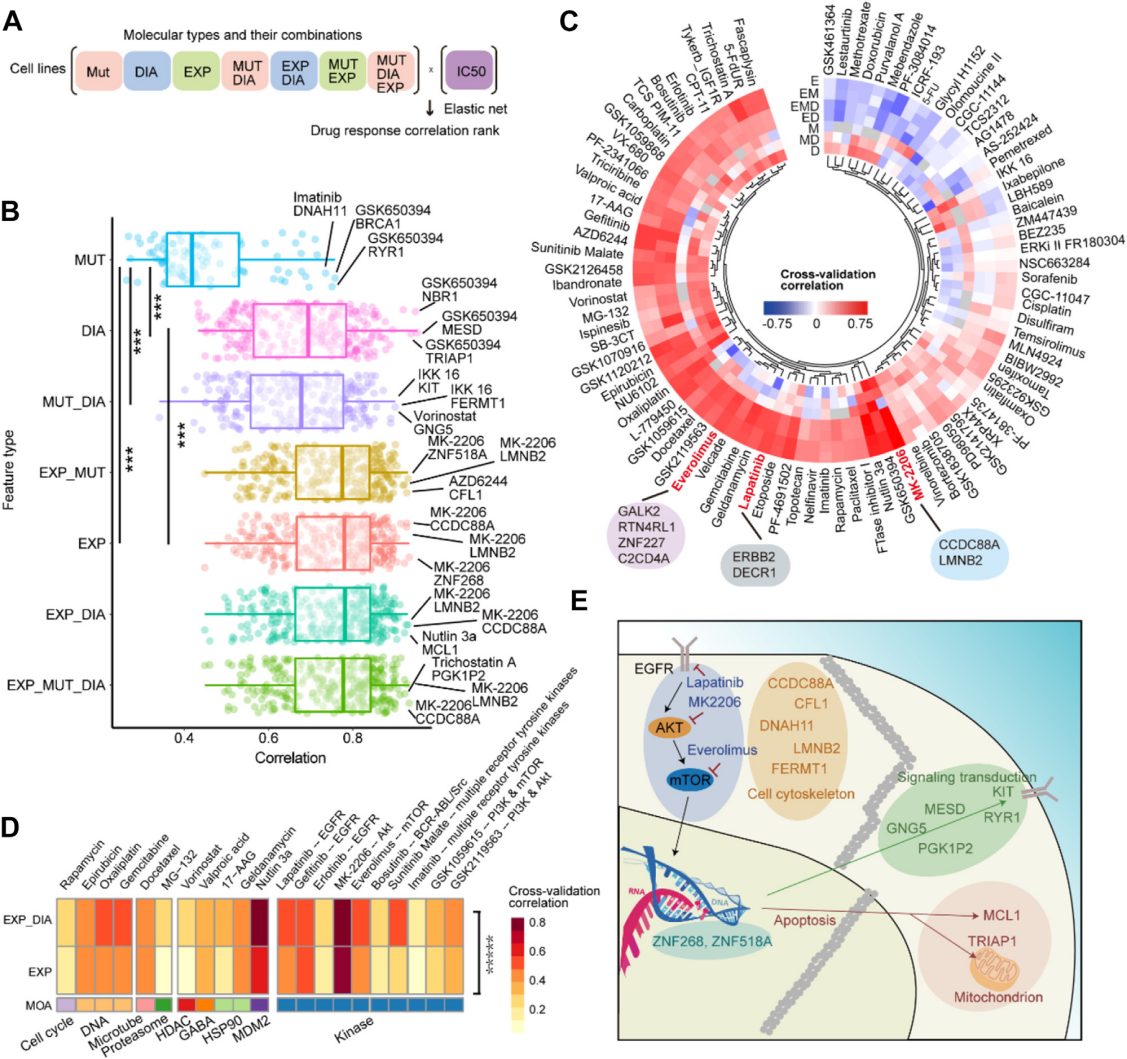
**Multi-omics analysis for drug response.***A*, workflow of our multi-omics analysis of the drug response by the elastic net algorithm. MUT (M): DNA mutation data (123 mutations); DIA (D) dataset (6091 proteins); EXP (E): transcriptomics dataset (29,140 transcripts). *B*, boxplot of drug response predictive power of different dataset combinations (y-axis). Each row represents one of the 89 valid elastic net models for the 90 drugs. *C*, Circos heatmap of the multi-omics results for each drug. *D*, predictive power comparison between the two omics datasets for 21 drugs (∗∗∗∗∗ represents *p-value* = 4.4 × 10∧-6 tested by paired *t* test). The color indicates the predictive power evaluated by Pearson correlation of cross-validation predicted and observed drug response values. The column contains the labels for the drug's mechanism of action (MOA) and the name of the drug. And the specific targeted kinases are annotated after the name of drugs. *E*, biological graph showing the protein biomarkers and pathways involved in the response to everolimus, lapatinib, and MK-2206 from the elastic net model.

We used the rcellminerElasticNet package (8), a wrapper around the glmnet R package and the elastic net algorithm, to explore how integrating various multi-omics datasets affected the response predictions for 90 drugs (5). Our analysis included the following instances: point mutations (123 mutations, supplemental Table S1*F*), RNAseq (29,140 mRNAs), proteomic data sets (6091 proteins), and RPPA (218 analytes) (Fig. 4*A*). For any drug and data combination, the prediction accuracy was evaluated using the Pearson correlation between the observed and the predicted drug responses. The overall predictive power of the DIA proteomic data was slightly lower than that of the RNA-seq data, but superior to that of the gene mutations (Fig. 4*B*). The relatively low predictive power of the mutation data might be attributable to the low numbers of mutations detected, which likely reflects the relatively low levels of single-nucleotide variants found in breast cancer (51).

We also displayed the multi-omics drug sensitivity prediction using a Circos plot (Fig. 4*C*); more information is provided in supplemental Table S2. This analysis revealed that the various multi-omic data types complement each other in predicting the response to certain drugs. Of note, the DIA proteomic data exhibited added value, relative to genomics and transcriptomics data, for multiple drugs, including lapatinib (HER2/EGFR inhibitor), MK-2206 (AKT1/2 inhibitor), and everolimus (mTOR inhibitor). The combination of DIA-based proteomic and transcriptomic data provided better predictive data-combo for 21 drugs (Fig. 4*D*). We then focused on the EGFR and Akt-mTOR signaling pathway which is crucial for many cancers including BC. Among the three EGFR inhibitors, Lapatinib exhibited the best performance. The molecules associated with the response to these three drugs (lapatinib, MK-2206, and everolimus) are listed in supplemental Table S2. To visualize the molecules involved in the response to these drugs, we compiled all the relevant proteins, as annotated by KEGG, into a diagram describing their interactions (Fig. 4*E*). These proteins are mainly involved in cell cytoskeleton, signal transduction, and apoptosis, indicating that these biological processes might be associated with the response to the respective drugs. In particular, AKT inhibitors (such as GSK650394) have been suggested as a treatment for BRCA1-deficient BCs (52). TRIAP1, an anti-apoptosis factor, is a marker of doxorubicin resistance (53). As CCDC88A can be phosphorylated by AKT to promote tumor proliferation (54), it might also be influenced by AKT inhibitors, such as MK-2206.

### Protein Networks Associated With Drug Response

**Fig. 5:**
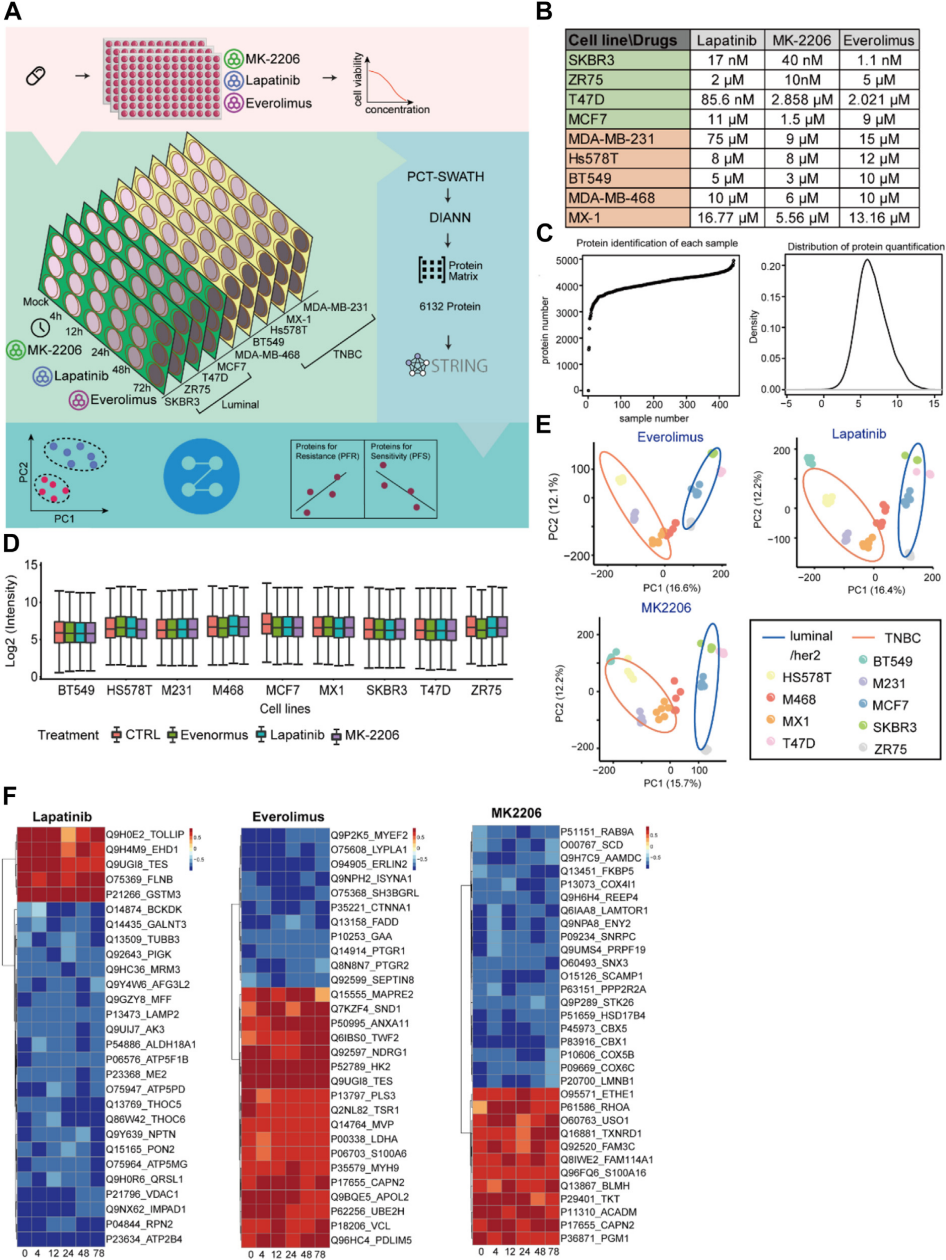
**Perturbation experiments on nine BC cell lines using lapatinib, everolimus, and MK-2206.***A*, workflow of our drug perturbation experiments on a set of BC cell lines. *B*, IC_50_ values of each cell line and for each drug. *C*, the number of identified proteins in each sample. *D*, density plot of the quantified protein intensities for each cell line. *E*, PCA plots of the different cell subtypes. *F*, heatmaps of the correlation values between the protein expression and IC_50_ values for the three drugs over time. The x-axis shows the time points (hrs), while the y-axis lists the clustered proteins. The proteins included in the heatmap are those with missing values in less than two time points, and a mean correlation coefficient >0.7.

Based on our multi-omics modeling of drug response predictions, we next investigated the effect of drug-induced perturbations on the proteomes of BC cell lines. In particular, we aimed to identify protein modules mediating the response to specific drug treatments and explore the mechanisms to reverse drug resistance. We focused on the three targeted therapy drugs with the strongest correlation of half-maximal inhibitory concentration (IC50) values with multi-omic data: lapatinib, MK-2206, and everolimus. We examined their effects on nine BC cell lines: SKBR3, T47D, MCF7, Hs578T, ZR75, BT549, MDA-MB-231, MDA-MB-468, and MX-1 (Fig. 5*A*). For each drug/cell line combination, we first determined the IC_50_ (Fig. 5*B*). The cell line was then treated with each drug at its IC_50_ concentration for 4, 12, 24, 48, and 72 h. Three biological replicates were generated, and lysates of the respective samples were analyzed by DIA-MS (Fig. 5*A*). A total of 5621 SwissProt proteins were identified in 432 samples with a 33.6% missing rate of 5621 proteins identified in all samples (Fig. 5, *C* and *D*; supplemental Table S3*A*). The high Pearson correlation value among the replicates, as shown in supplemental Fig. S5*A* demonstrated the high reproducibility of the experiment and MS measurement. In the PCA analysis of each drug, the luminal lines were well separated from the TNBC samples, indicating that the variance in BC subtypes introduces more significant variations than the drug perturbations (Fig. 5*E*).

We next analyzed the correlation between protein levels and IC50 values in each cell line at various time points after drug treatment (supplemental Table S3, *B*–*D*). In Figure 5*F*, the proteins shown in red, including five proteins for lapatinib, 18 proteins for everolimus, and 12 proteins for MK-2206, were positively correlated with IC50 values, therefore they potentially contribute to drug resistance. In contrast, there are several proteins in blue, including 23 proteins for lapatinib, 11 proteins for everolimus, and 20 proteins for MK-2206. Their expression levels were negatively correlated with the IC50 values, therefore they might be correlated with drug sensitivity. Among the proteins that positively correlated with drug resistance, several have been reported to be associated with therapy response. For example, hexokinase 2 (HK2) is required for tumor progression (55) and interacts with mTOR leading to tamoxifen resistance (56). CAPN2, which is known to promote castration-resistant prostate cancer invasion *via* AKT/mTOR (57), has been reported to be upregulated in TNBC patients compared with HER2-positive BCs (58). Together, these proteins might be associated with the sensitivity of BC after lapatinib, everolimus, and/or MK-2206 treatment.

### Dysregulated Metabolism of Perturbed TNBC Cell Lines

**Fig. 6:**
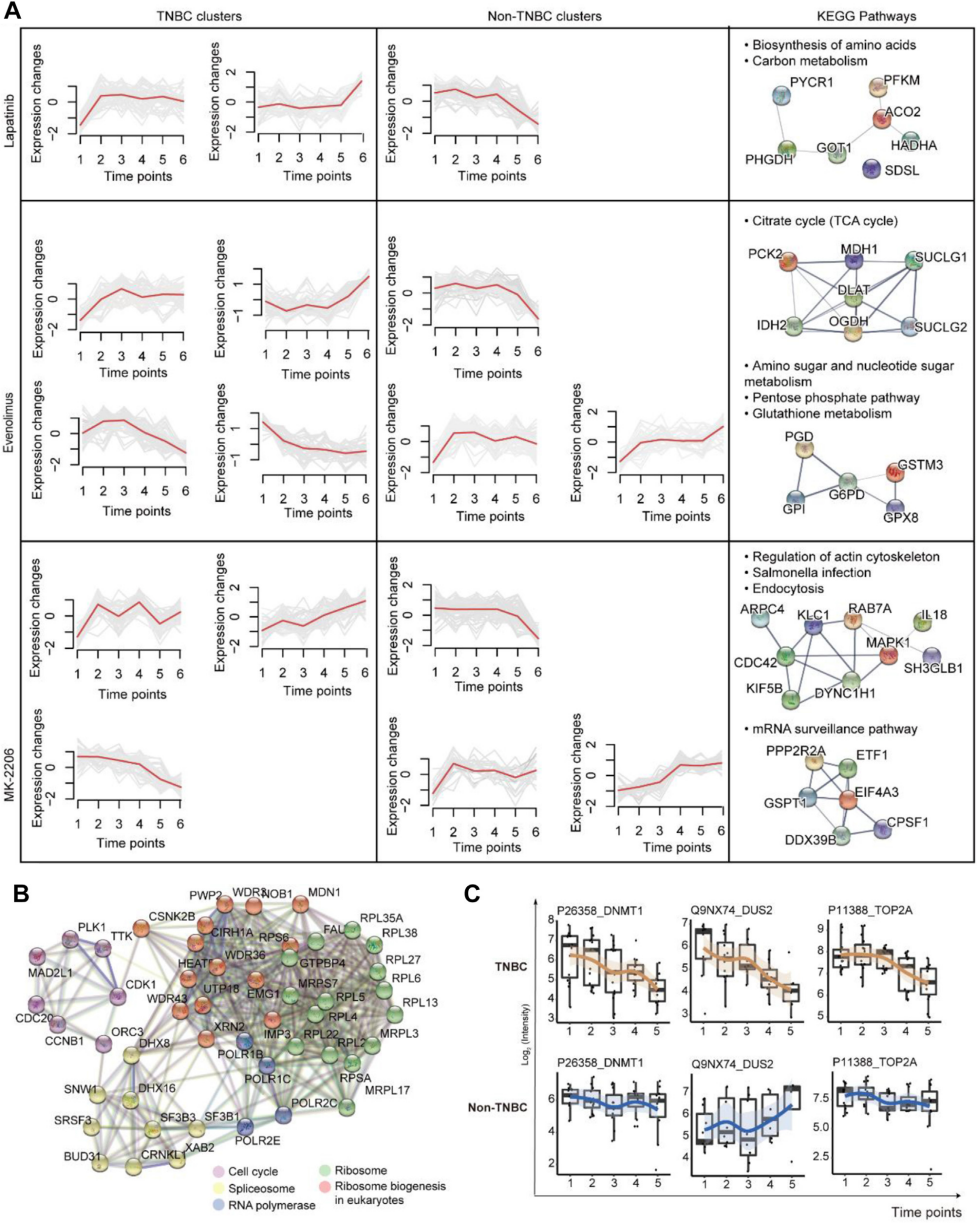
**Expression trend comparisons in TNBC and non-TNBC cell lines.***A*, time series of protein expressions showing opposite trends in the NBC and non-TNBC cell lines after treatment with lapatinib, everolimus, or MK-2206. Left: Mfuzz clustering over six time points (0, 4, 12, 24, 48, and 72 h), right: the filtered KEGG pathways. *B*, network analysis of the DEPs between 0 and 72 h in the TNBC cell lines (B-H adjusted *p-value* < 0.05, fold change > 1.5 or < 0.67). *C*, the relative abundance of four representative proteins across the TNBC and the non-TNBC cell lines after being treated with MK-2206. X-axis: drug treatment time; y-axis: log2-scaled intensity.

To compare the protein patterns that differed in drug response between TNBC and non-TNBC cell lines, we used Mfuzz and differential expression analysis. We identified 252 proteins with the opposite dynamic patterns between TNBC and non-TNBC cell lines (see Experimental Procedures, Fig. 6*A* and supplemental Table S3*E*). Aligning the proteomic data with biological pathways, the results showed that lapatinib and everolimus induced distinct metabolic responses in TNBC and non-TNBC cell lines, respectively (Fig. 6*A*). This result is in line with previous studies highlighting metabolism as a hallmark of cancer (59). In particular, we observed the induction of the tricarboxylic acid cycle (TCA), amino sugar and the nucleotide sugar metabolism, the pentose phosphate pathway, and glutathione metabolism in TNBC lines (Fig. 6*A*). The TCA cycle occurs in mitochondria, which were reported to be dysregulated at baseline in metastatic, compared with non-metastatic, TNBC (60). The mTOR signaling pathway can regulate mitochondrial function (61), potentially contributing to the metabolic changes observed following everolimus treatment. By contrast, actin cytoskeleton and mRNA surveillance pathways were altered after MK-2206 treatment (Fig. 6*A*).

We identified over 2000 DEPs between the pre-treatment and the last post-treatment time point for each of the three drugs in each cell line (supplemental Fig. S5*B*). To identify the key protein determinants contributing to the response to lapatinib, MK-2206, and everolimus in TNBC cells, we compared the persistently dysregulated proteins (PDPs) after the drug treatment in TNBC and non-TNBC cell lines. We found 483 PDPs in over 60% of TNBC cell lines treated with MK-2206, while there were only 173 PDPs for everlolimus and 172 PDPs for lapatinib (supplemental Fig. S5*C*). Hence, MK-2206 treatment induced the most significant differences between the TNBC and non-TNBC cell lines at the proteome level. The set of affected proteins mainly included proteins involved in ribosome biogenesis, RNA polymerase, spliceosome, and the cell cycle (Fig. 6*B*). Among these, DNA (cytosine-5)-methyltransferase-1 (DNMT1) was significantly downregulated only in the TNBC cell lines (Fig. 6*C*). AKT has been reported to reduce DNA methylation *via* modulating the phosphorylation of DNMT1, and DNMT1 inhibition could also reduce the activity of AKT signaling (62). Future study of this dataset may identify similar drug combination opportunities that exploit adaptive changes in protein level.

Two additional proteins were downregulated in the TNBC cell lines but did not change or increased in abundance in the non-TNBC cell lines after MK-2206 treatment, including tRNA-dihydrouridine (20) synthase [NAD(P)+]-like (DUS2) and DNA topoisomerase 2-alpha (TOP2A) (Fig. 6*C*). DUS2 is involved in tRNA modification and affects the stability of mtRNA (63). Separately, a previous study has verified that reduced expression of TOP2A led to extensive resistance to a broad spectrum of drugs (64). Notably, TOP2A has been reported to have key functions in chromosome segregation and is a predictor of response for many cancer therapies (8, 65).

Collectively, the proteins and related pathways that we identified through analyses of these large datasets lay the foundation for future studies into potential TNBC treatment. Our observations are further reinforced by previous work on the implications of these proteins and pathways in cancer development.

## Conclusion

We collected high-quality proteomics data on 76 BC cell lines with four replicates using PCT-DIA and integrated these data with genomics and transcriptomics for drug sensitivity prediction. We observed that proteomic data improved the sensitivity prediction of multiple drugs in BC. Our analysis identified 38 proteins specifically expressed in TNBC cell lines, which were validated using independent data. Our DIA dataset contributes to the effective prediction of drug response in multi-omics modeling experiments. Additionally, we performed time-course analyses investigating protein dynamics to identify proteins and pathways that are specifically dysregulated in TNBC lines and might yield insight into adaptive drug resistance in TNBC. Although lapatinib and everolimus elicited few changes in TNBC cells, MK-2206 resulted in significant changes including ones involving RNA modification and cell cycle pathways. Our data showed significant changes in DUS2, DNMT1, and TOP2A following MK-2206 treatment in TNBC. These observations warrant future investigation to explore their suitability as drug targets or biomarkers.

### Limitations

Although this multi-omics study included a relatively large number of samples, as well as biological and technical replicates, it did not include phosphorylation data. However, this limitation does not compromise the relevance and accuracy of our main findings. The primary focus of this publication was to report on this rich proteomic dataset and conduct an initial, exploratory bioinformatic analysis of the dataset. It is outside the scope of the intended work, and therefore a limitation, to conduct in-depth experimental validation of reported biomarkers; this work is left for future study.

## Data and Materials Availability

The 76 breast cancer cell DIA data are deposited in Pride (PXD004701), the protein matrix is included as Supplemental Table S1B. The DIA data of cell lines drug perturbation is deposited in iProX (IPX0001825001), and the protein matrix is included as Supplemental Table S3A.

## Supplemental Data

This article contains supplemental data.

## Conflict of interest

T. G. and Y. Z. are shareholders of Westlake Omics Inc. R. A. holds shares in Biognosys. W. G. are employees of Westlake Omics Inc. The remaining authors declare no competing interests.

## Supplemental Data

Supplemental Table S3

Supplemental Figures S1 **Analysis of reproducibility and batch effects.***A*, the distribution of peptide lengths in all samples, including biological replicates (rep 1, 2, 3) and technical replicates (rep4). *B*, the distribution of peptide counts in all samples, including biological replicates (rep 1, 2, 3) and technical replicates (rep4). *C*, the coefficient of variation (CV) between the replicates of DIA data. The proteins were classified into four groups according to the interquartile range of protein intensity distribution. From the lowest to the highest intensity, the abundance level 1 is the top 25%, 2 is from 25% to 50%, 3 is from 50% to 75%, 4 is the last 25%. The median CV of the four abundance levels are 0.00, 0.17, 0.10 and 0.04, respectively. *D*, distribution of protein abundances in all samples. *E*, PCA plots of all samples among four groups using total 6091 proteins. *F*, PCA plots of all samples among four groups using total all 6091 proteins after batch effect removal. **Supplemental Figure S2**. **Correlation between mRNA and protein data for selected individual proteins.** Expression correlations of seven proteins between mRNA and RPPA, RPPA and DIA, and mRNA and DIA. The Pearson correlation was tested by Pearson correlation test. **Supplemental Figure S3**. **Correlation between mRNA and protein data for the components of selected protein complexes.***A* and *B*, the 10 most correlated protein complexes at transcript and the protein level. The Pearson correlation was tested by Pearson correlation test. **Supplemental Figure S4**. **Validation of the 38-protein signatures in four independent datasets capable of distinguishing the TNBC and the non-TNBC cell lines.***A*, PCA plot of the differentially expressed transcripts (DETs) between basal and non-basal cell lines (B-H adjusted *p-*value < 0.05, fold change > 1.5 or < 0.67). *B*, Venn diagram showing the overlapping differentially expressed proteins (DEPs) between Asleh *et al.* dataset and our dataset (BR76). *C*, the protein expression of the 96 overlapping DEPs in the Asleh *et al.* dataset and BR76. *D*, validation of the 38-protein signatures using the Lawrence *et al.* (50) dataset. *E*, validation of the 38-protein signatures using the Bouchal *et al.* (17) dataset. *F*, validation of the 38-protein signatures using the Lapek *et al.* (15) dataset. **Supplemental Figure S5**. **Quality control analysis of the drug perturbation experiment of over nine BC cell lines.***A*, the number of identified proteins *B*, the number of DEPs between different time points and control sample without treatment (B-H adjusted *p-*value < 0.05, fold change > 1.2). *C*, the numbers of persistently dysregulated proteins (PDPs) in different cell lines treated with three drugs. The PDPs were identified by overlapping the proteins from the persistently dysregulated clusters of mfuzz and those showing persistently positive or negative correlation between the protein expression and time points after treatment.
