# Supplementary figures and images for: Proteomic Dynamics of Breast Cancer Cell Lines Identifies Potential Therapeutic Protein Targets
Source: Mol Cell Proteomics. 2023 Jun 19;22(8):100602. doi: 10.1016/j.mcpro.2023.100602 (PMC10392136; doi:10.1016/j.mcpro.2023.100602)

**Figure S1**

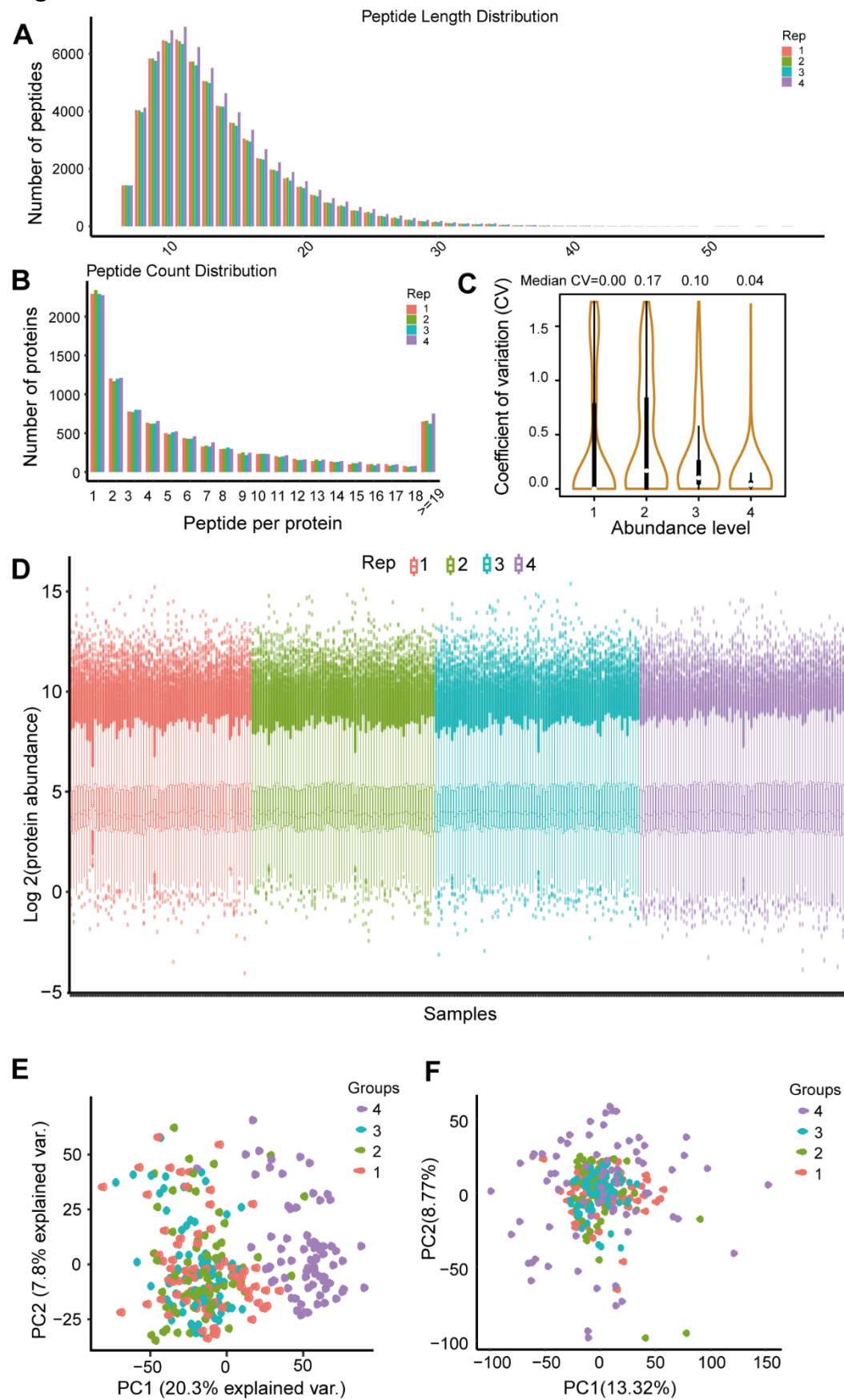

**Figure S2**

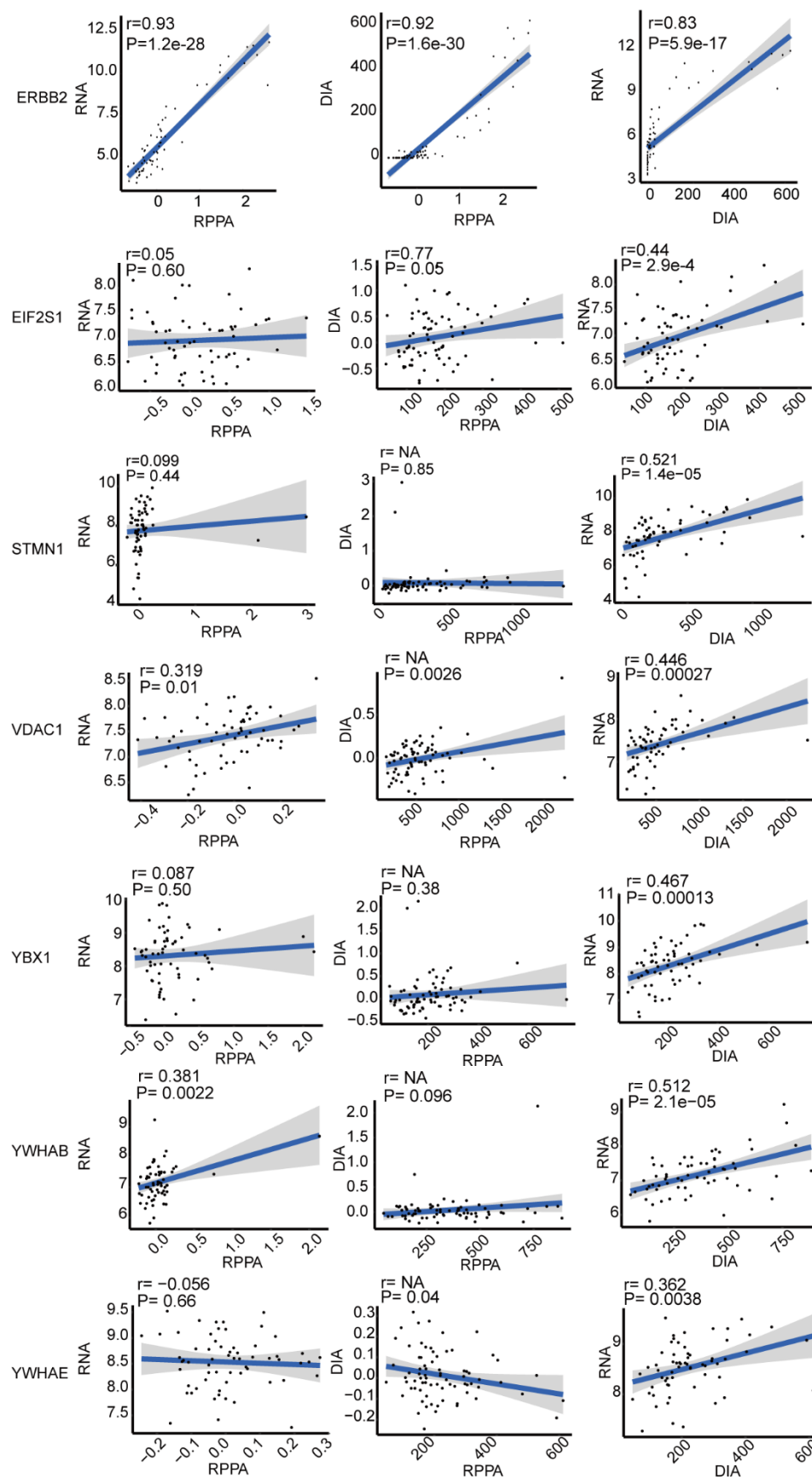

**Figure S3**

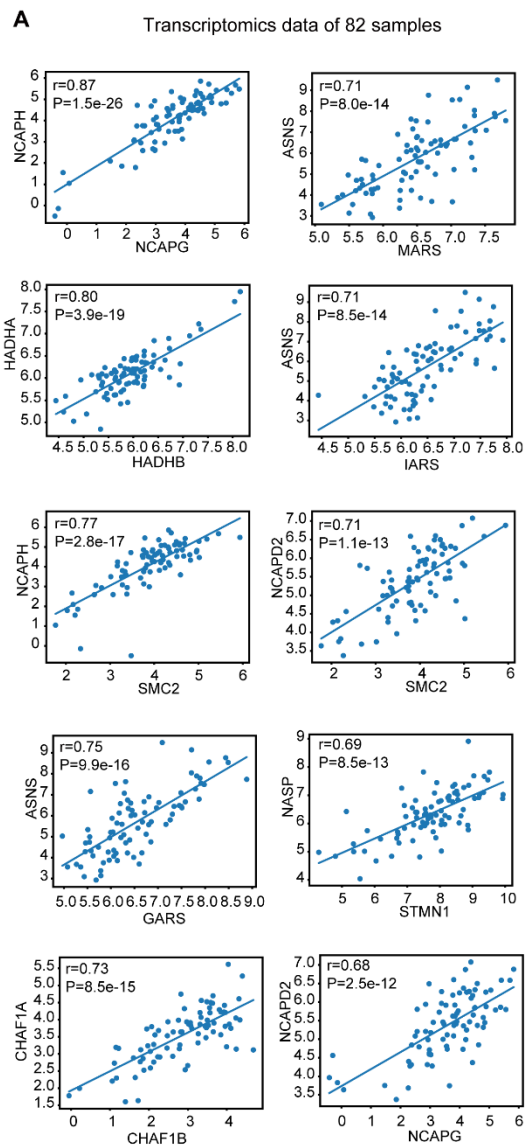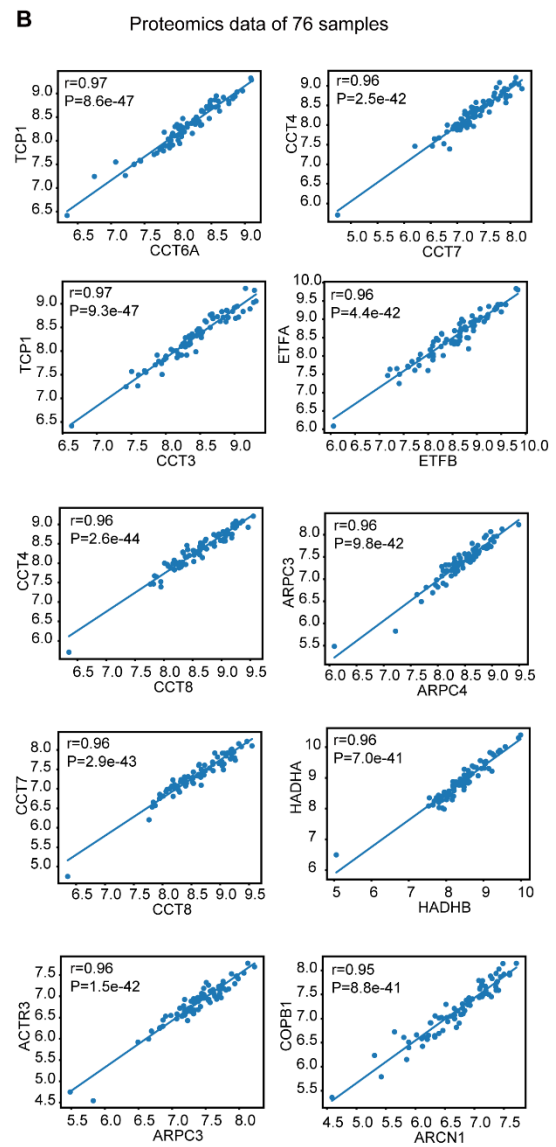

**A**

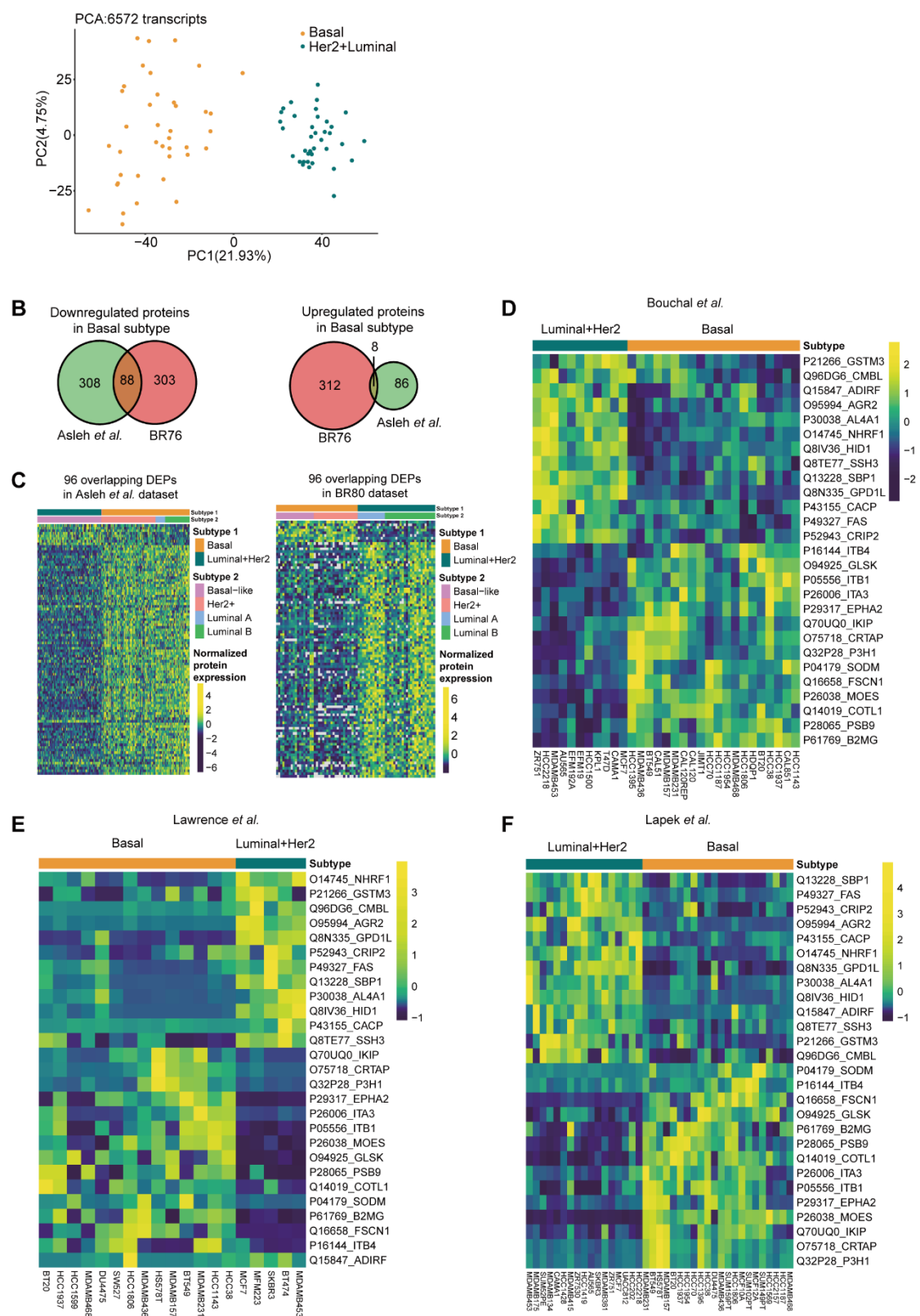

Figure S5

A

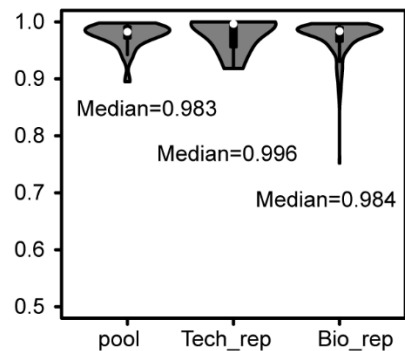

B

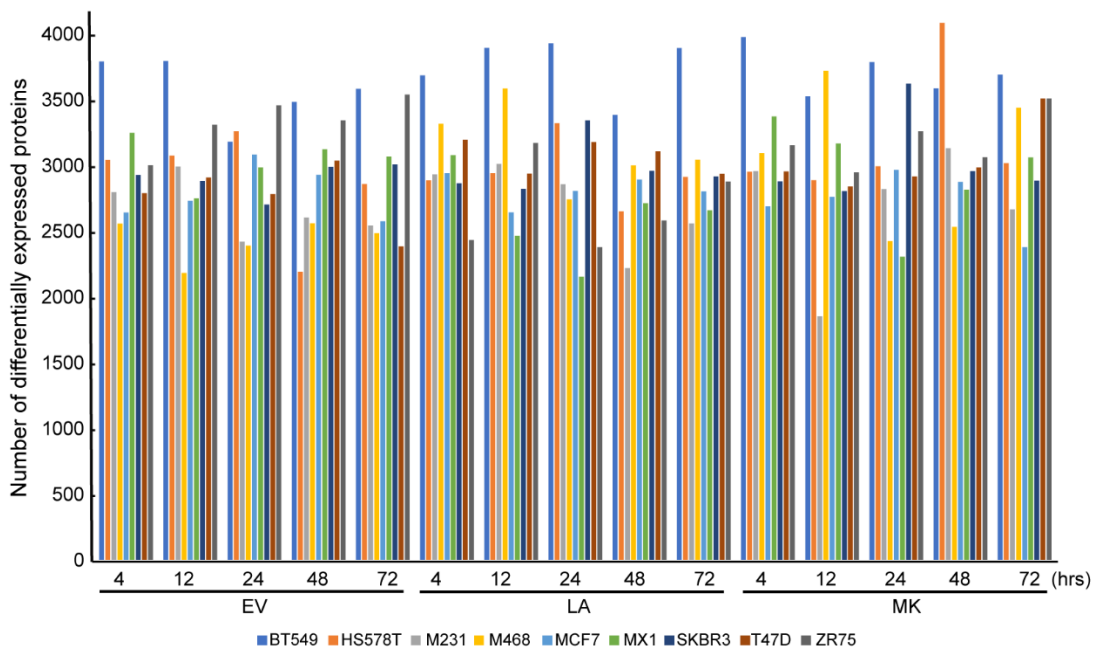

C

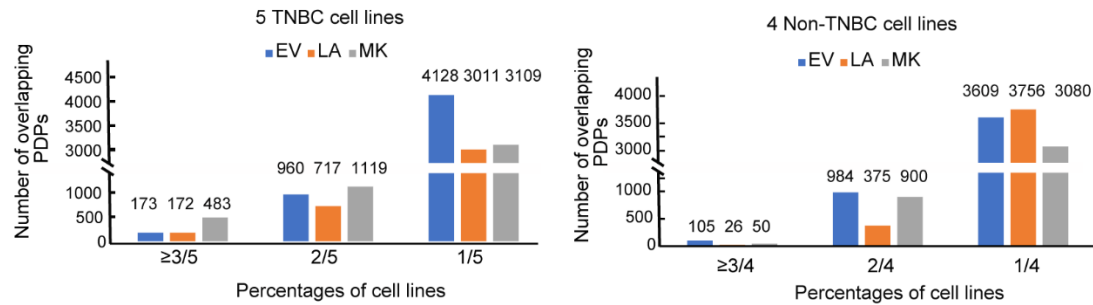

Supplement: Supplemental Figures S1 — Analysis of reproducibility and batch effects.A, the distribution of peptide lengths in all samples, including biological replicates (rep 1, 2, 3) and technical replicates (rep4). B, the distribution of peptide counts in all samples, including biological replicates (rep 1, 2, 3) and technical replicates (rep4). C, the coefficient of variation (CV) between the replicates of DIA data. The proteins were classified into four groups according to the interquartile range of protein intensity distribution. From the lowest to the highest intensity, the abundance level 1 is the top 25%, 2 is from 25% to 50%, 3 is from 50% to 75%, 4 is the last 25%. The median CV of the four abundance levels are 0.00, 0.17, 0.10 and 0.04, respectively. D, distribution of protein abundances in all samples. E, PCA plots of all samples among four groups using total 6091 proteins. F, PCA plots of all samples among four groups using total all 6091 proteins after batch effect removal. Supplemental Figure S2. Correlation between mRNA and protein data for selected individual proteins. Expression correlations of seven proteins between mRNA and RPPA, RPPA and DIA, and mRNA and DIA. The Pearson correlation was tested by Pearson correlation test. Supplemental Figure S3. Correlation between mRNA and protein data for the components of selected protein complexes.A and B, the 10 most correlated protein complexes at transcript and the protein level. The Pearson correlation was tested by Pearson correlation test. Supplemental Figure S4. Validation of the 38-protein signatures in four independent datasets capable of distinguishing the TNBC and the non-TNBC cell lines.A, PCA plot of the differentially expressed transcripts (DETs) between basal and non-basal cell lines (B-H adjusted p-value < 0.05, fold change > 1.5 or < 0.67). B, Venn diagram showing the overlapping differentially expressed proteins (DEPs) between Asleh et al. dataset and our dataset (BR76). C, the protein expression of the 96 overlapping DEPs in [file mmc4.pdf]
